# Comparative Analysis of the Gut Microbiota of Adult Mosquitoes From Eight Locations in Hainan, China

**DOI:** 10.3389/fcimb.2020.596750

**Published:** 2020-12-15

**Authors:** Xun Kang, Yanhong Wang, Siping Li, Xiaomei Sun, Xiangyang Lu, Mamy Jayne Nelly Rajaofera, Yajun Lu, Le Kang, Aihua Zheng, Zhen Zou, Qianfeng Xia

**Affiliations:** ^1^Key Laboratory of Tropical Translational Medicine of Ministry of Education and School of Tropical Medicine and Laboratory Medicine, Hainan Medical University, Haikou, China; ^2^CAS Center for Excellence in Biotic Interactions, University of Chinese Academy of Sciences, Beijing, China; ^3^State Key Laboratory of Integrated Management of Pest Insects and Rodents, Institute of Zoology, Chinese Academy of Sciences, Beijing, China

**Keywords:** Hainan, mosquitoes, midgut microbiome, bacterial community, 16S rRNA

## Abstract

The midgut microbial community composition, structure, and function of field-collected mosquitoes may provide a way to exploit microbial function for mosquito-borne disease control. However, it is unclear how adult mosquitoes acquire their microbiome, how the microbiome affects life history traits and how the microbiome influences community structure. We analyzed the composition of 501 midgut bacterial communities from field-collected adult female mosquitoes, including *Aedes albopictus*, *Aedes galloisi*, *Culex pallidothorax*, *Culex pipiens, Culex gelidus*, and *Armigeres subalbatus*, across eight habitats using the HiSeq 4000 system and the V3−V4 hyper-variable region of 16S rRNA gene. After quality filtering and rarefaction, a total of 1421 operational taxonomic units, belonging to 29 phyla, 44 families, and 43 genera were identified. *Proteobacteria* (75.67%) were the most common phylum, followed by *Firmicutes* (10.38%), *Bacteroidetes* (6.87%), *Thermi* (4.60%), and *Actinobacteria* (1.58%). The genera *Rickettsiaceae* (33.00%), *Enterobacteriaceae* (20.27%), *Enterococcaceae* (7.49%), *Aeromonadaceae* (7.00%), *Thermaceae* (4.52%), and *Moraxellaceae* (4.31%) were dominant in the samples analyzed and accounted for 76.59% of the total genera. We characterized the midgut bacterial communities of six mosquito species in Hainan province, China. The gut bacterial communities were different in composition and abundance, among locations, for all mosquito species. There were significant differences in the gut microbial composition between some species and substantial variation in the gut microbiota between individuals of the same mosquito species. There was a marked variation in different mosquito gut microbiota within the same location. These results might be useful in the identification of microbial communities that could be exploited for disease control.

## Introduction

Mosquitoes (Culicidae) are vectors of many human diseases. To satisfy the reproductive nutritional needs, females required repeated blood meal from a host. This can result in the initial acquisition and later transmission of pathogens (Wang et al., 2017). Mosquitoes are the main vectors of several kinds of pathogenic factors of various human infectious diseases, such as O’nyong-nyong, Zika virus, dengue virus, chikungunya virus, and West Nile virus (Barrett and Higgs, 2007; Fauci et al., 2016; Tsetsarkin et al., 2016; Weaver et al., 2016; Rezza et al., 2017). The transmission of mosquito-borne pathogens involves interactions between vectors, pathogens, and vertebrate hosts. Once the mosquito has ingested a blood meal from an infected host, the pathogen first invades the midgut epithelium. It then moves through the hemolymph to secondary tissues, such as the trachea and fat body, and finally, infects the salivary glands. At this stage, the pathogen contaminates mosquito saliva and is injected into a vertebrate host when the mosquito takes a blood meal (Wang and Jacobs-Lorena, 2013; Sim et al., 2014; Wang et al., 2017). Through this process, the mosquito is infectious and can transmit the pathogen. However, the mosquito midgut has factors that might impede successful transmission of the pathogen and also affect the biology of the host (Wang et al., 2011; Baia-da-Silva et al., 2019). These factors include components of the mosquito innate immune system, such as lectins, bacteria-derived cytolysins (hemolysins), antimicrobial peptides, peroxidase, proteases, digestive enzymes, secondary metabolites, nitric oxide, and prophenoloxidase (Ramirez et al., 2012; Sim et al., 2014; Wang et al., 2019).

Culture-dependent and culture-independent approaches have been used to study the microbial communities in mosquito midguts. Midgut bacteria of mosquitoes have often coevolved with their hosts, and many of these endosymbionts cannot be cultured on standard microbiological media (Gandotra et al., 2018). Gut bacteria that can be cultured illustrate symbiotic associations. Some culturable gut symbionts have been functionally characterized in their host insects. For pathogen control, some studies have used genetic engineering techniques on symbiotic bacteria. Symbiotic bacteria are used to deliver anti-pathogen effector molecules to the midgut lumen and render host mosquitoes refractory to pathogen infection (Wang et al., 2012). The composition and diversity of gut microbiota vary within and between different mosquito species. Microbiota are influenced by host diet, developmental stage, population location, larval environment and pathogen infection (Muturi et al., 2016a). Studies on the microbial communities from different locations and species of mosquitoes can improve understanding of the gut microbiota. There is also a need to extend microbiome studies to other mosquito-borne disease systems that may be used for disease control.

Hainan province, in southern China, is separated by a strait from Guangdong Province and situated between latitudes 18°10’ N and 20°10’ N and longitudes 108°37’ E and 111°03’ E. The Island has a convergence of tropical and subtropical areas with a climate and environment favorable for mosquitoes. Most Hainan studies have focused on the surveillance and control of the vectors of Zika virus and malaria (Wang et al., 2014; Yasri and Wiwanitkit, 2017). The molecular characterization of species and the effects of insecticides or antifungal agents have also been investigated (Sun et al., 2017; Li et al., 2018; Ruiling et al., 2018). Little is known about bacteria in the mosquito midguts or microbial symbionts. To gain a better understanding of the diversity and function of mosquito gut microbiota in Hainan province, we conducted microbiome studies. We identified bacteria in the midguts of mosquitoes from eight geographical areas in Hainan to evaluate differences in diversity, composition, and structure of midgut microbiota between collection sites and mosquito species.

## Materials and Methods

### Mosquito Collection and Morphology Identification

**Figure 1:**
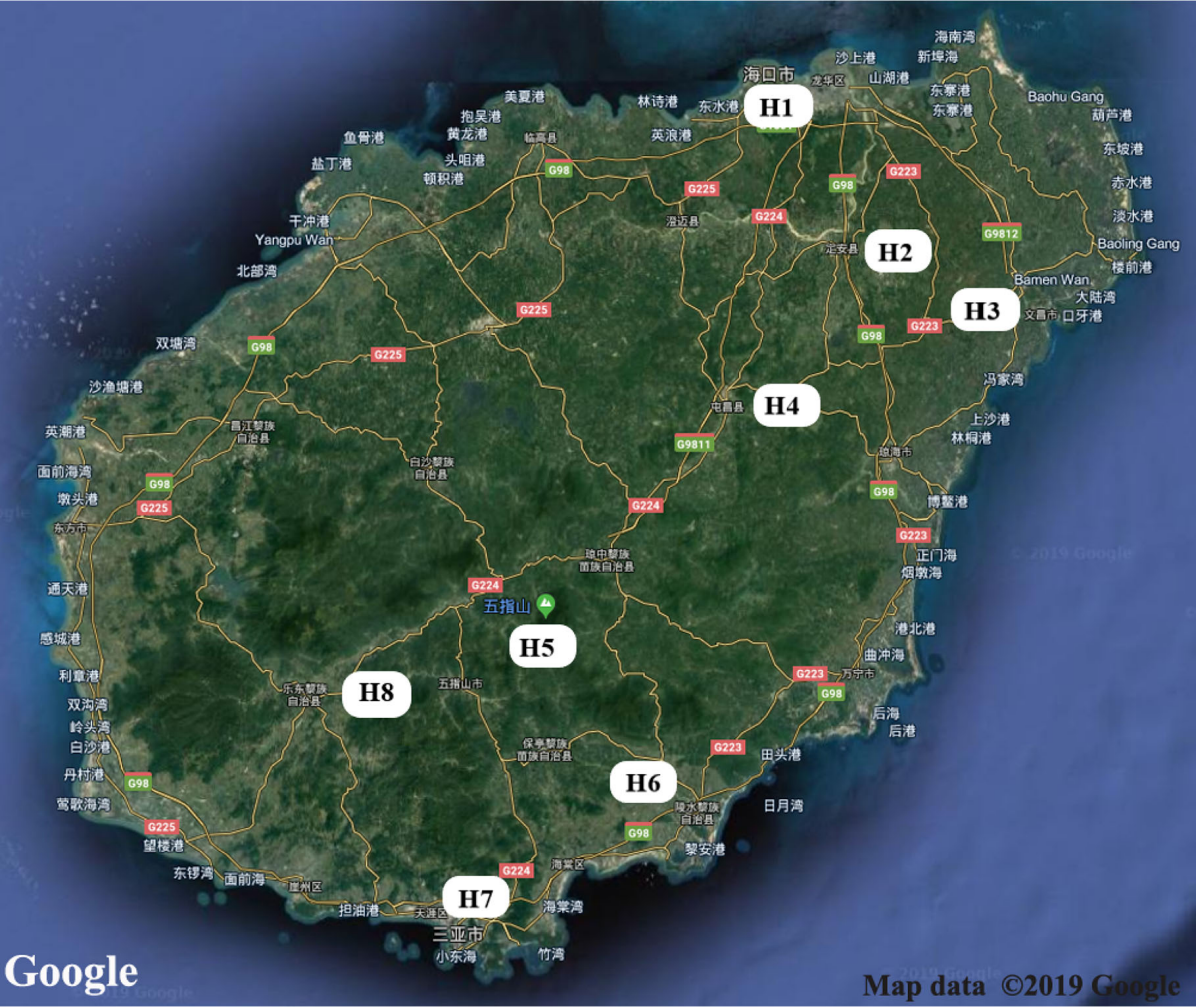
Map of Hainan province showing locations of the eight collection study sites. H1 = Haikou, Urbanna; H2 = Dingan, Country; H3 = Wenchang, Urbanna; H4 = Tunchang, farm; H5 = Wuzhishan, montane; H6 = Lingshui, montane; H7 = Sanya, Urbanna; H8 = Ledong, Urbanna.

Adult mosquitoes were collected in 2018 (July 1 to September 18) from eight sites in Hainan province, China (H1-H8: H1 = Haikou; H2 = Dingan; H3 = Wenchang; H4 = Tunchang; H5 = Wuzhishan; H6 = Lingshui; H7 = Sanya; H8 = Ledong) (**Figure 1**). Mosquitoes were collected using nets, aspirators, light traps coupled with a CO_2_ source, and human landings. The trap sites we selected include households, the woods, residential areas, near the pool, parks, and construction sites. Specifically, a light trap was placed at the sheltered site away from light and about 1.5 m above the ground. The light was on, and surveillance was performed at night from 1 h before sunset to 1 h after sunrise (Li et al., 2019). During the day, adult mosquitoes were collected in using human-landing catches. Larvae and pupae were sampled with pipettes and sieves. Adult mosquitoes from immature samples were collected after eclosion. Mosquitoes were identified to species using the keys of Wilke (Wilke et al., 2016), and returned to the laboratory. Species identifications were confirmed using a diagnostic PCR assay based on DNA barcode analysis (Ashfaq et al., 2014).

### Molecular Identification of Mosquitoes

For species identification, two legs were removed from each female adult and transferred to a well pre-loaded with 200 μl of 95% ethanol in a 96-well microplate. PCR was performed to amplify the 5’ cytochrome c oxidase subunit 1 (cox1) region of the mitochondrial DNA (Kumar et al., 2007) using the forward primer LCO1490 (5′-GGTCAACAAATCATAAAGATATTGG-3′) and reverse primer HCO2198 (5′-TAAACTTCAGGGTGACCAAAAAATCA-3′) (Steyn et al., 2016). Each amplification was performed in a 50 µl volume that included 1 µl of DNA template, 2.5 µl of the forward and reverse primers, 25 µl of 2×Taq PCR Mastermix (KT201), and 21.5 µl of Millipore water. The PCR cycle included initial denaturation 95°C for 3 min, 35 cycles each of denaturation 95°C for 30 s, primer annealing 55°C for 30 s, and primer extension 72°C for 1.5 min followed by 10 min extension at 72°C and storage at 4°C. PCR products were run in 1.0% agarose gel stained with ethidium bromide (EB) and visualized in a gel imaging system.

### Midgut Dissection and DNA Extraction

All mosquitoes were kept in 1.5 ml Eppendorf tubes at –80˚C until DNA extraction, and all were processed at the same time to limit batch effects (Krajacich et al., 2018). The microbial communities were identified from a pooled sample. After molecular identification, a total of 11–30 midguts were dissected for each mosquito species, and the samples were evenly distributed, and three groups were repeated. Each female mosquito was surface sterilized in 75% ethanol for 10 min, then rinsed three times in sterile PBS solution. Midguts were dissected and stored individually at −20°C until processing. DNA was extracted using the DNeasy Blood & Tissue Kit following manufacturer instructions (Qiagen, Valencia, CA, USA). Isolated DNA was reconstituted in 50 μl of ddH_2_O and stored at −20°C for further processing. A 50 μl aliquot of the DNA isolate was used to build a microbiome library for Illumina HiSeq sequencing at BGI Shenzhen (Shenzhen, China).

### Sample Processing and 16S rRNA Gene Library Preparation

A total of 501 midguts, from different mosquito species at eight sites, were processed (**Table 1**). The hypervariable regions V3−V4 of the bacterial 16S rRNA were amplified with primers 338F (5′-ACTCCTACGGGAGGCAGCAG-3′) and 806R (5′-GGACTACHVGGGTWTCTAAT-3′) (Zhang et al., 2018) containing multiplex identifier sequences (Wu et al., 2016). The PCR reaction of purified DNA from each midgut sample was set up as follows: each 25 μl reaction consisted of microbial DNA (5 ng/μl) 2.5 μl; amplicon PCR reverse primer (1 μmol/L) 5 μl; amplicon PCR forward primer (1 μmol/L) 5 μl; 2× KAPA HiFi Hot Start Ready Mix 12.5 μl. The plate was sealed, and PCR was performed in a thermal instrument (Applied Biosystems 9700, USA) including an initial denaturation step at 95°C for 3 min, followed by 25 cycles of denaturing at 95°C for 30 s, annealing at 55°C for 30 s, elongation at 72°C for 30 s, and a final extension at 72°C for 5 min (Wu et al., 2016). The PCR products were checked using electrophoresis in 1.0% (w/v) agarose gels in TBE buffer (Tris, boric acid, EDTA) stained with EB and visualized under UV light. For PCR products, the jagged ends of DNA fragments were converted into blunt ends using T4 DNA polymerase, Klenow Fragment, and T4 Polynucleotide Kinase. Then we added an ‘A’ base to each 3’ end to ease addition of adapters. Afterwards, short fragments were removed by Ampure beads. For genomic DNA, we used a fusion primer with dual index and adapters for PCR and short fragments were also removed by Ampure beads. In both cases, only the qualified Illumina library was used for sequencing on HiSeq 4000 system.

**Table 1:** Number of mosquito samples processed for midgut bacteria.

Site	Mosquito species	Number of midgut samples	
		Initial	Final
Lingshui (LS)	*Aedes galloisi* (AGA)	34	30
	*Aedes albopictus* (AAL)	39	30
Tunchang (TC)	*Culex pallidothorax* (CPA)	25	18
	*Aedes albopictus* (AAL)	30	27
Haikou (HK)	*Culex pipiens* (CPI)	42	30
	*Culex gelidus* (CGE)	13	11
	*Aedes albopictus* (AAL)	35	30
	*Armigeres subalbatus* (ASU)	32	30
Dingan (DA)	*Aedes albopictus* (AAL)	31	30
	*Culex pallidothorax* (CPA)	16	15
Wenchang (WC)	*Armigeres subalbatus* (ASU)	32	30
	*Aedes albopictus* (AAL)	33	30
	*Culex pallidothorax* (CPA)	19	14
Sanya (SY)	*Aedes albopictus* (AAL)	34	30
	*Armigeres subalbatus* (ASU)	30	29
Wuzhishan (WZS)	*Culex pipiens* (CPI)	29	27
	*Armigeres subalbatus* (ASU)	31	30
	*Aedes albopictus* (AAL)	32	30
Ledong (LD)	*Aedes albopictus* (AAL)	32	30
		569	**501**

Each female mosquito was surface sterilized in 75% ethanol for 10 min, then rinsed three times in sterile PBS solution. A total of 11–30 midguts were dissected for each mosquito species, and the samples were evenly distributed, and three groups were repeated.

### Data Processing, Filtering, and Fragment Assembly

To obtain more accurate results, raw data were pre-processed to get clean data using the following procedure: (1) Truncation of sequence reads lacking an average quality of 20 over a 30 bp sliding window based on the Phred algorithm, and trimming of reads with less than 75% of their original length, as well as paired reads; (2) Removal of reads contaminated by adapter (default parameter was 15 bases overlapped by reads and an adapter with more than three mismatched bases); (3) Removal of reads with an ambiguous base (N base), and its paired reads; (4) Removal of reads with low complexity (default: reads with 10 consecutive same bases). If the two paired-end reads overlapped, the consensus sequence was generated by the Software FLASH (Fast Length Adjustment of Short reads v1.2.11) (Magoc and Salzberg, 2011). The criteria were as follows: Minimal overlapping length was 15 bp and the allowable reads had an error outed using Quantitative Insights into Microbial Ecology v1.9.1 (QIIME) (Caporaso et al., 2010) quality filters.

### OTU Clustering, Annotation, and Analysis

High-quality sequences were grouped in OTUs with an open-reference selection method at 97% similarity threshold (Navas-Molina et al., 2013) using USEARCH v 7.0.1090 (Edgar, 2013). UCHIME v4.2.40 (Edgar et al., 2011) was used to remove the chimeric sequences by PCR amplification from the OTU representative sequence. All tags are mapped to each OTU representative sequence using USEARCH GLOBAL (Wang et al., 2007). The tag number of each OTU in each sample was summarized by an OTU abundance table. OTU representative sequences were classified using the Ribosomal Database Project (RDP) Classifier v.2.2 (https://rdp.cme.msu.edu/index.jsp) (Wang et al., 2011) and trained on the Greengenes database. The parameters had confidence values of 0.8 if the length of tags was ≥ 250 bp; otherwise 0.5 confidence values were used as the cutoff. The taxonomic assignment was based on comparison with the closest matched sequences on the Greengene (v201305) (http://greengenes.lbl.gov/cgi-bin/nph-index.cgi) database. We removed unassigned OTUs and we removed OTUs that were not assigned to the target species. The filtered OTUs were used for downstream processing.

### Bacterial Diversity Analysis

To assure that a randomly selected amplicon from a sample was previously sequenced, we used Good’s coverage index (the number of OTUs sampled more than once divided by the total number of OTUs) to estimate sequencing depths implemented in Mothur (http://www.mothur.org/wiki/Calculators). Rarefaction curves were drawn using the mean indices calculated with extracted tags, in R software, as a function of the number of randomly sampled tags with the vegan package (Huber et al., 2015).

Alpha diversity (α-diversity) describes within sample diversity, including species richness and evenness. Alpha diversity indices were calculated by Mothur v1.31.2. Corresponding rarefaction curves and box/bar plots were drawn using R software. The midgut bacterial communities’ indices included species richness (observed species, Chao1, ACE) and species diversity (Simpson index and Shannon). For calculation of each index we used formulas in http://www.mothur.org/wiki/Calculators. Differential analysis among groups was done using the alpha diversity indices. The Wilcoxon Rank-Sum Test was used for comparisons between two groups, while the Kruskal-Wallis Test was used for multi-group comparisons (Schloss et al., 2009). The analyses were performed using R v3.1.1 software.

Alpha diversity describes within sample diversity while beta diversity (β-diversity) describes the similarity of two samples. QIIME (Caporaso et al., 2010). The β-diversity of gut bacterial communities among locations was estimated using the Bray-Curtis dissimilarity index (Hernandez-Garcia et al., 2018). The unweighted Bray-Curtis index considers only phylogenetic richness and the weighted index considers both relative abundance and phylogenetic richness (Lozupone et al., 2010). Unweighted and weighted UniFrac Principal Coordinate Analysis (PCoA) plots were used to visualize microbial community structure relationships. PCoA (Mohammadi Shahrestani et al., 2015) was applied to taxon abundance profiles, including phylum, class, order, family, genus and species profiles. The ‘ade4’ package in R v3.1.1 was used for PCoA analysis.

The difference in the abundance of microbial communities between the two groups of samples was tested statistically, and the FDR (false discovery rate) was used to assess the significance of the difference. Metastats software (http://metastats.cbcb.umd.edu/) (default) or R software (Rank sum test, Fisher’s exact test, Chi-square test, T-test and Variance test) for significant differences were used for analysis. The correction of the p-value is performed *via* p-adjust in the R package (v3.1.1), and the correction method is BH (Benjamini - CHochberg) (White et al., 2009)

## Results

### Mosquito Species From Different Sampling Areas

We identified six mosquito species: *Aedes albopictus*, *Aedes galloisi*, *Culex pallidothorax*, *Culex pipiens, Culex gelidus*, and *Armigeres subalbatus*. These mosquito species had distinct geographical distributions. *Ae. albopictus* was found in all eight collection sites; *Ar. subalbatus* was found in Haikou, Wenchang, Sanya, and Wuzhishan; *Cx pallidothorax* was found in Tunchang, Dingan, and Wenchang; *Cx. pipiens* was found in Haikou, Wuzhishan, Lingshui, and Haikou; *Ae. galloisi* and *Cx. gelidus* were found in Lingshui and Haikou, respectively (**Table 1**). These results suggest that many mosquito species in Hainan are widely distributed and have regional characteristics.

### Midgut Bacterial Species Composition Across Mosquito Species

We analyzed the V3-V4 hyper-variable region of the 16S rRNA gene to estimate the composition of bacterial communities in 501 midguts of field-collected adult female mosquitoes. A total of 5,154,464 raw reads (**Table S1**) (Mean ± SE = 62859.32 ± 7871.44 per mosquito midgut sample) were obtained from the 501 mosquito samples that were sequenced. After quality control, 4,585,398 clean reads (**Table S1**) remained for subsequent analyses.

**Figure 2:**
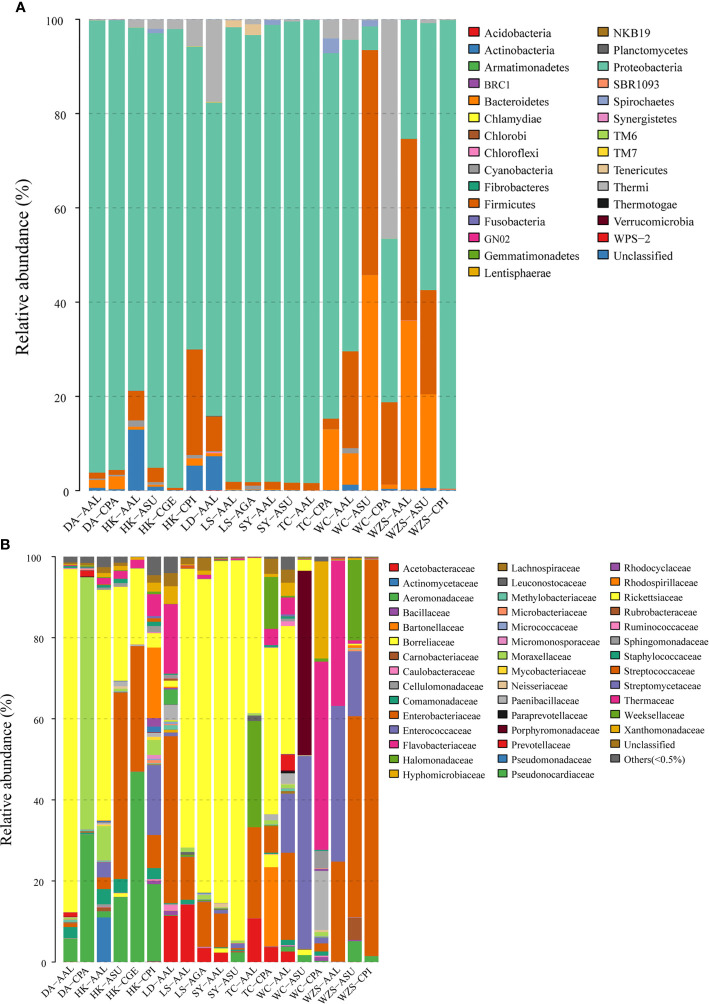
Mean relative abundances of bacterial phyla **(A)** and families **(B)** associated with six mosquito species at different sites and collection dates. Families with abundance less than 0.5% were pooled together as “Other.” (DA, Dingan; HK, Haikou; LD, Ledong; LS, Lingshui; SY, Sanya; TC, Tunchang; WC, Wenchang; WZS, Wuzhishan; AAL, *Aedes albopictus*; ASU, *Armigeres subalbatus*; CGE, *Culex gelidus*; AGA, *Aedes galloisi*; CPA, *Culex pallidothorax*; CPI, *Culex pipiens*).

Filtered tags were clustered into OTU (Operational Taxonomic Units) with 97% similarity. These sequences were clustered into 1,421 bacterial OTUs belonging to 29 phyla, 44 families, and 43 genera (including one unclassed and others) (**Table S2**). To avoid obvious deviations in the microbial richness, OTUs with relative abundance of more than 0.5% were selected for comparative analysis. Most of the sequences came from *Proteobacteria* (75.67%), including *Alphaproteobacteria* (38.84%), *Gammaproteobacteria* (35.43%), *Betaproteobacteria* (1.38%) (**Figure 2A**; **Table S3**). Other observed phyla included *Firmicutes* (10.38%), *Bacteroidetes* (6.87%), *Thermi* (4.60%), *Actinobacteria* (1.58%), *Spirochaetes* (0.35%), *Cyanobacteria* (0.28%), *Tenericutes* (0.20%), *Chloroflexi* (0.02%), *TM7* (0.01%), *Acidobacteria* (0.009%), and Unclassified (0.002%) (**Figure 2A**; **Table S3**).

Families with the highest OTU abundance included *Rickettsiaceae* (33.00%), *Enterobacteriaceae* (20.27%), *Enterococcaceae* (7.49%), *Aeromonadaceae* (7.00%), *Thermaceae* (4.52%), and *Moraxellaceae* (4.31%) (**Figure 2B**; **Table S3**). At the genus level, the most abundant microorganisms in the guts of mosquitoes were *Wolbachia* (31.89%), Unclassified (25.90%), *Enterococcus* (7.48%), *Thermus* (4.52%), *Acinetobacter* (4.28%), *Escherichia* (2.74%), *Enterobacter* (2.61%), *Dysgonomonas* (2.40%), *Swaminathania* (2.33%), *Thorsellia* (2.16%), *Serratia* (1.76%), *Providencia* (1.33%), and *Rickettsia* (1.10%). Other genera with relative abundance less than 0.5% were identified (**Table S3**; **Figure S1**).

*Rickettsiaceae* occurred in high abundance among all mosquito species except *Ae. albopictus* and *Cx. pipiens* from Wuzhishan. However, their amount was fewer in *Cx. pipiens*, *Cx. pallidothorax*, and *Cx. gelidus* compared to the remaining mosquito species. Among the *Rickettsaceae*, *Wolbachia* was more prevalent and abundant in the guts of *Ae. albopictus* and *Ae. galloisi* from most areas but had low abundance in *Ar. subalbatus*, *Cx. pipiens and Ae. albopictus* from Wuzhishan. *Enterobacteriaceae* was more common among *Ae. albopictus* from Ledong, Tunchangand, and Wenchang; it occurred in high abundance in the guts of a few individuals of *Ar. subalbatus* and *Cx. pipiens* from Wuzhishan (**Figure 2B**; **Table S3**). At the genus level, there were many unclassified, especially in *Ae. albopictus* and *Cx. pipiens* mosquitoes in Wuzhishan, which accounted for 60.30% and 99.14% of the total (**Table S3**).

**Figure 3:**
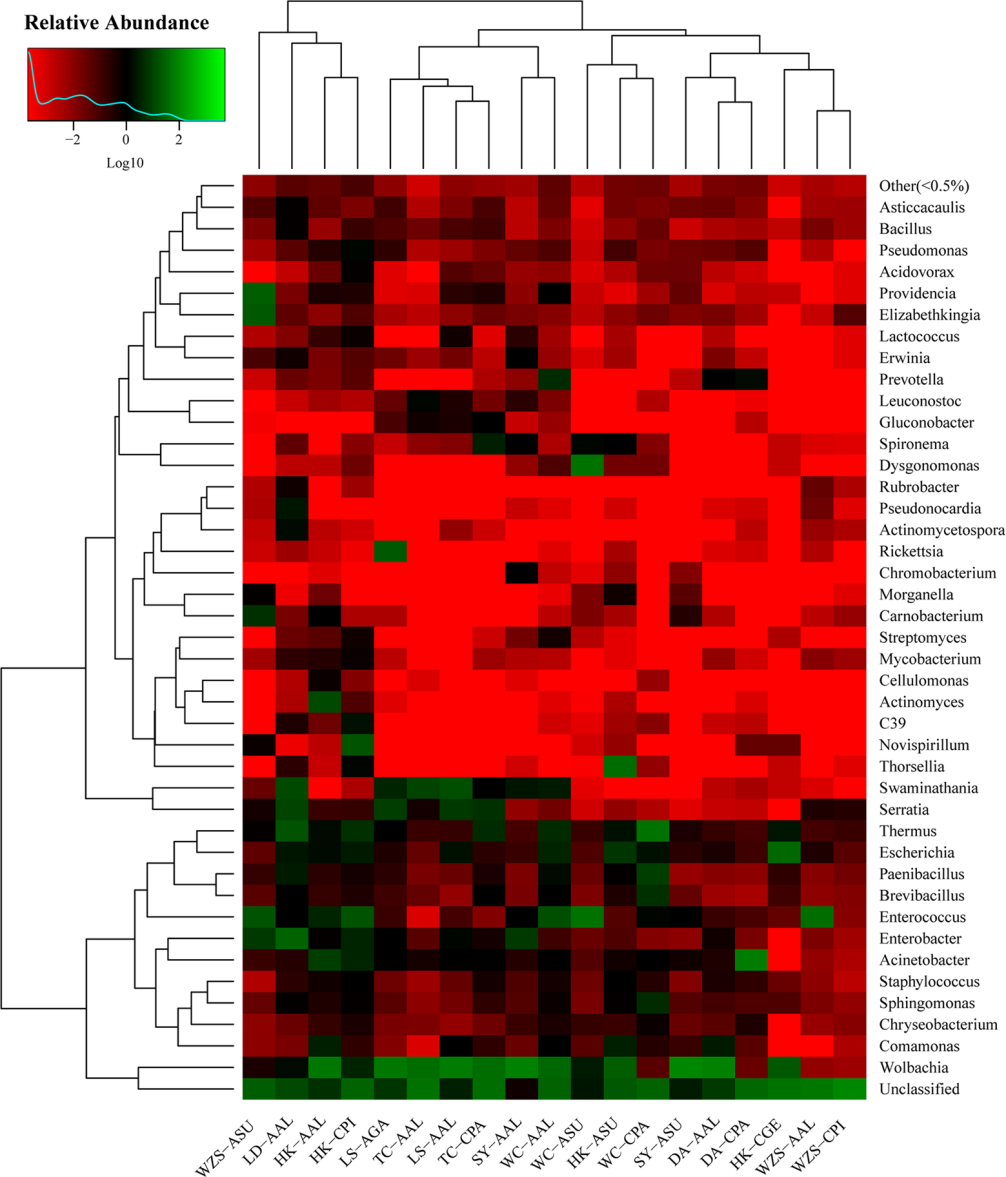
Heatmap in log scale depicting the gut bacterial community of mosquito midguts obtained with open reference OTU picking methods. Green colors represent high abundance and red colors represent low abundance; black indicates absence.

The gut bacterial communities were different in composition and abundance among the locations of all mosquito species. The shifts of microbial compositions in dominant genera are shown on the heat map (**Figure 3**).

### Diversity of Mosquito Microbiota

**Figure 4:**
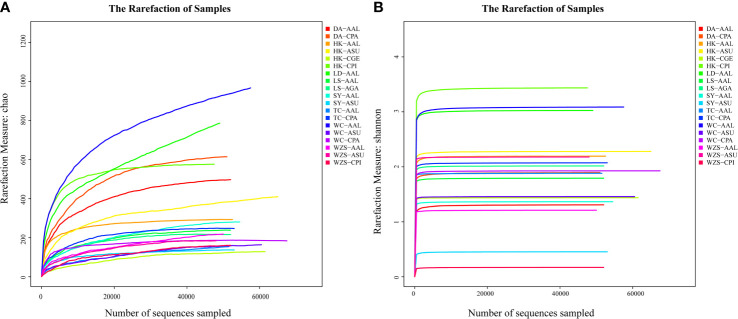
Rarefaction analysis of observed richness Chao **(A)** and Shannon index **(B)** within individual mosquitoes.

Rarefaction analysis showed that the microbial richness and diversity varied among individual mosquitoes, since the libraries were sampled and prepared at different depth (**Figures 4A, B**). Rarefaction curves for some individual mosquitoes did not plateau, indicating the potential for unrecovered rare bacterial taxa. To determine whether bacterial diversity and richness varied significantly among mosquito species and study sites, we computed the alpha diversity indices along with 95% confidence intervals (**Table 2**).

**Table 2:** Diversity and richness (mean and 95% confidence limits) of the midgut bacterial communities of six mosquito species from eight sites in Hainan province.

Site	Species	Group	Sobs	Chao1	Ace	Shannon	Simpson
Dingan	*Ae. albopictus*	DA-AAL	226.33 (149.00–363.00)	259.04 (172.88–382.13)	273.58 (170.52–382.83)	1.22 (1.12–1.38)	0.52 (0.50–0.53)
Dingan	*Cx. pallidothorax*	DA-CPA	286.83 (214.00–378.00)	320.33 (238.41–417.94)	323.52 (243.14–430.24)	1.87 (1.68–2.09)	0.24 (0.22–0.26)
Haikou	*Ae. albopictus*	HK-AAL	160.33 (111.00–228.00)	172.48 (125.88–234.00)	170.48 (127.48–230.85)	2.04(0.60–3.67)	0.38 (0.07–0.38)
Haikou	*Ar. subalbatus*	HK-ASU	206.33 (184.00–220.00)	233.05 (202.05–246.46)	232.79 (209.89–248.60)	1.65(0.85–2.16)	0.40 (0.20–0.74)
Haikou	*Cx. pipiens*	HK-CPI	208.83 (71.00–287.00)	219.68(91.65–287.67)	219.45(100.95–288.11)	2.48 (1.09–3.73)	0.29 (0.07–0.56)
Ledong	*Ae. albopictus*	LD-AAL	312.00 (213.00–386.00)	360.79 (229.92–468.83)	357.80 (227.23–440.25)	2.32 (1.48–2.94)	0.28 (0.16–0.45)
Lingshui	*Ae. albopictus*	LS-AAL	104.00 (78.00–119.00)	115.33(87.07–138.09)	114.46(89.20–129.45)	1.65 (1.25–2.37)	0.28 (0.15–0.38)
Lingshui	*Ae. galloisi*	LS-AGA	113.00 (97.00–144.00)	125.39 (103.07–160.24)	126.11(104.59–158.68)	1.91 (1.68–2.20)	0.23 (0.19–0.29)
Sanya	*Ae. albopictus*	SY-AAL	77.11 (44.00–103.00)	91.01(53.00–109.18)	89.72(51.25–110.91)	1.01 (0.73–1.56)	0.46(0.27–0.58)
Sanya	*Ar. subalbatus*	SY-ASU	69.00 (62.00–83.00)	76.27(66.20–93.11)	75.04(66.04–92.12)	0.41 (0.35–0.52)	0.87 (0.81–0.90)
Tunchang	*Ae. albopictus*	TC-AAL	72.00 (59.00–90.00)	101.87(93.20–118.33)	122.38(99.24–160.86)	1.46 (1.30–1.59)	0.30(0.23–0.39)
Tunchang	*Cx. pallidothorax*	TC-CPA	138.33 (101.00–162.00)	164.94(127.40–189.00)	165.80 (133.44–184.32)	1.50(1.09–2.00)	0.41 (0.23–0.61)
Wenchang	*Ae. albopictus*	WC-AAL	287.33 (87.00–602.00)	315.67 (122.00–658.95)	316.13 (131.35–638.66)	2.34(0.87–4.17)	0.26(0.05–0.61)
Wenchang	*Ar. subalbatus*	WC-ASU	59.43 (27.00–112.00)	87.39(38.25–135.21)	125.40 (48.34–185.58)	0.98 (0.69–1.43)	0.50(0.33–0.63)
Wuzhishan	*Ae. albopictus*	WZS-AAL	139.33 (113.00–170.00)	230.57(185.53–272.14)	266.28(237.11–246.39)	1.21 (1.17–1.27)	0.33 (0.33–0.34)
Wuzhishan	*Ar. subalbatus*	WZS-ASU	84.86 (29.00–129.00)	138.49(53.00–245.67)	214.59(69.95–341.78)	1.28 (0.68–1.63)	0.38(0.27–0.73)
Wuzhishan	*Cx. pipiens*	WZS-CPI	96.33 (95.00–98.00)	171.69 (145.17–185.50)	231.15 (146.47–278.99)	0.17 (0.11–0.20)	0.95 (0.94–0.97)
LAB	*Ae. albopictus*	LAB-AAL	155.00 (98.00–252.00)	193.68(151.2–267.79)	206.50 (151.19–264.20)	1.30 (1.06–1.64)	0.48 (0.39–0.52)
LAB	*Ae.aegypti*	LAB-AAE	195.33 (78.00–307.00)	210.00 (111.00–315.00)	210.69 (115.40–313.40)	2.05 (0.25–3.10)	0.40 (0.10–0.92)

**Figure 5:**
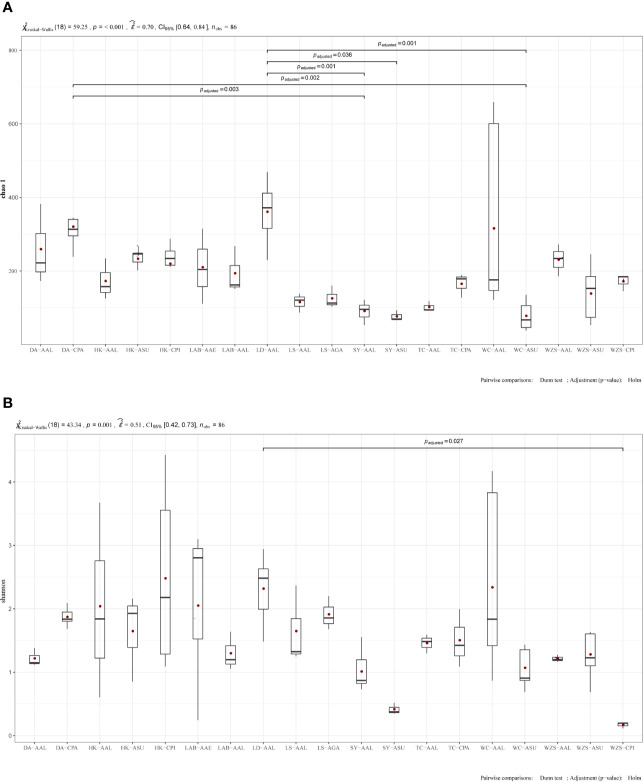
Boxplot representation of observed species. Boxplots show distribution of bacteria between mosquito samples categorized under different locations and mosquito species. Boxplot representation of chao1 **(A)** and Shannon diversity **(B)**. Significant differences between the groups were investigated by Pairwise comparisons of means: Dunn test; Adjustment (p−value): Holm. Species richness is represented by the number of bands. Box plots show median value and minimum and maximum values. Black lines indicate medians and the value is shown in the figure. Values from all statistical tests are available in additional **Table 2**.

The midgut bacterial communities’ indices included species richness (observed species, Chao1, ace) and species diversity (Simpson index and Shannon) (**Table 2**). Chao1 and ace of species richness means the number of species in the community, regardless of the abundance of each species in the community. Shannon diversity and Simpson diversity of species diversity influenced by species richness and species evenness in the sampled community. The species richness was estimated by Chao1, and the difference was statistically significant in alpha diversity. Bacterial richness was significantly higher among *Ae. albopictus* from Ledong compared to *Ae. albopictus* from Sanya (Kruskal-Wallis test: p = 0.001, Fig 5A), *Ar. subalbatus* from Sanya (p = 0.036, Fig 5A) and *Ae. albopictus* from Wenchang. (p = 0.001, Fig 5A). In addition, *Cx. pallidothorax* from Dingan had significantly higher bacterial richness compared to *Ae. albopictus* from Sanya (p=0.003, Fig 5A) and *Ar. subalbatus* from Wenchang (p = 0.002, **Figure 5A**). The species diversity was estimated by Shannon, significantly different in *Ae. albopictus* from Dingan compared to *Cx. pipiens* from Wuzhishan (p = 0.027, **Figure 5B**). Species diversity was observed in other groups, but the difference was not statistically significant (**Figure 5B**). Values from all statistical tests are available in additional **Table 2**.

### Variation of Midgut Bacterial Communities Across Mosquito Species

We used several different distance metrics to assess differences in bacterial community profiles between species and geographical locations. The QIIME analysis based on Bray-Curtis distances revealed a significant difference in microbial communities among the six mosquito species and eight locations. To visualize the results, a PCoA plot, based on an unweighted Unifrac distance matrix, was used to depict the differences in the composition of the gut microbiota from different locations.

Three PCoA coordinates using unweighted Unifrac (uwU) distance percent variation explained PCo1 - 26.75%, PCo2 - 12.20%, PCo3 - 10.41% of the total variation, respectively (**Figure S2**). Significant differences were found in midgut bacterial β-diversity among communities from different localities. For instance, *Cx. pipiens* from Wuzhishan and Haikou (**Figure S2 A**); *Ae. albopictus* from Wenchang and Wuzhishan (**Figure S2 B**); *Ae. albopictus* from Ledong and *Ar. subalbatus* from Sanya were not tightly clustered (**Figure S2C**). No significant difference was found among the bacterial communities of the same localities for the midgut of different mosquitoes (**Figure S2A**). These differences and similarities were mainly due to the presence or absence of unclassified taxa and geographical location, respectively.

**Figure 6:**
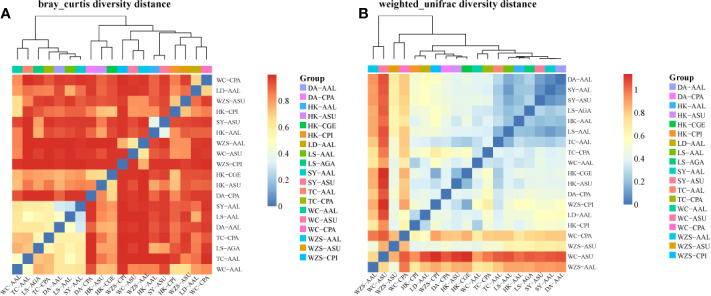
Matrix heatmap of Bray-Curtis distances **(A)** and Weighted unifrac Beta diversity **(B)** between microbial communities of six mosquito species from eight areas.

A diversity matrix heatmap was generated based on Bray-Curtis distances (**Figure 6A**) and Weighted unifrac β-diversity (**Figure 6B**). Ordination based on this matrix heatmap showed not only clear separation but also clustering of the samples, which reflects both the diversity and the similarity between the gut microbiota. The R statistics ranged from 0.00 to 1.00. The closer the value is to 1, the greater the difference between samples. Values closer to 0, indicate more similar samples. Bonferroni correction for multiple comparisons revealed 19 significant pairwise comparisons (**Figure 6**). The community structure of microbiota of *Cx. pipiens* collected from Wuzhishan was substantially different from that of *Cx. pipiens* from Haikou (0.979), *Ae. galloisi* from Lingshui (0.994), *Ae. albopictus* from Ledong (0.986), Lingshui (0.994), Sanya (0.997) and Wenchang (0.984) (**Figure 6A**; **Table S4**). The community structure of microbiota of *Ae. albopictus* from Tunchang was substantially different from that of *Ae. albopictus* from Haikou (0.989), *Cx. pallidothorax* from Dingan (0.997) and Wenchang (0.996), *Ar. Subalbatus* from Sanya (0.988) and Wenchang (0.996) (**Figure 6A**; **Table S4**). A similar pattern of microbiota was observed between *Ae. albopictus* from Sanya and *Ae. albopictus* from Lingshui (0.100) and *Ar. subalbatus* from Sanya (0.093). In addition, *Ar. subalbatus* from Sanya, *Ae. albopictus* from Tunchang and *Cx. gelidus* from Haikou had a similar pattern of microbiota compared to *Ae. albopictus* from Haikou (0.202), *Ae. albopictus* from Lingshui (0.174) and *Cx. pipiens* from Wuzhishan (0.145). There was little separation between the microbiota of *Ae. albopictus* from Sanya and Dingan (0.0659) (**Figure 6B**; **Table S4**).

## Discussion

Substantial interactions can occur between resident or introduced arthropods and invasive pathogens. The midgut bacteria of mosquitoes play an important role in vector parasite interactions (Dong et al., 2009). Many studies have reported microbial diversity in insect guts but few studies have reported the microbial populations associated with mosquito midguts in Hainan. We conducted microbial inventories of different species of mosquitoes by sequencing the 16S rRNA gene, and we systematically analyzed bacteria from different locations by evaluating 16S rRNA contents. These findings add to the limited knowledge of the microbiota of different mosquito species and show how geographical location or host habitat can influence the composition and diversity of mosquito microbiota.

We reported the diversity of midgut bacteria of six species of mosquito. These were *Ae. albopictus*, *Cx. pipiens*, *Cx. gelidus*, *Ar. subalbatus*, *Cx. pallidothorax*, *Ae. galloisi* (**Table 1**) collected from eight sites (**Figure 1**) in Hainan, including urban areas (Haikou, Sanya and Wenchang), rural areas (Ledong and Dingan), farms (Tunchang), and virgin forests (Lingshui and Wuzhishan). We characterized and compared the midgut bacterial communities, many of which are vectors of medical, veterinary, and wildlife significance. *Ae. albopictus* and *Ar. subalbatus* were the dominant species in the eight sites on Hainan Island. *Ae. albopictus* is a vector of the dengue virus and chikungunya virus (Zouache et al., 2012). *Ar. subalbatus* is a vector of the filarial worm *Brugia pahangi* (Aliota et al., 2010). There were few consistent differences in the composition of gut microbiota among the different mosquito species. There was great intraspecific variation in the bacterial taxa present indicated by the dominance of one to three bacterial OTUs.

The bacterial community in mosquito midguts was dominated by five main phyla, and the microbial communities in the mosquito midguts were similar at family and genus levels across eight locations. These five bacterial phyla are commonly reported in the guts of mosquitoes and other insects (Zouache et al., 2012). The *Proteobacteria* are highly diverse and contain a variety of species that are adapted to a wide range of environments. Its dominance in mosquito midguts is well established (Muturi et al., 2016b; Strand, 2018). However, *Proteobacteria* occurred in low abundance in *Ae. albopictus* from Wenchang and *Ar. Subalbatus* from Wuzhishan. The percentage of *Actinobacteria* was significantly lower (1.58%) than in a previous study (Muturi et al., 2017; Oliveira et al., 2018).

The functions of the major bacterial genera identified in this study are unclear. *Wolbachia* (*Rickettsiaceae*) are intracellular and maternally inherited micro-organisms widespread in arthropods (Muturi et al., 2016c). They induce several reproductive disorders such as cytoplasmic incompatibility in their hosts, parthenogenesis and feminization to facilitate their spread into host populations (Blagrove et al., 2012). Studies on mosquito endosymbionts showed that *Wolbachia* (*Rickettsiaceae*) occur in most populations of the *Cx. pipiens* species complex. This complex consists of *Cx. pipiens* and *Cx. quinquefasciatus* (Diptera: Culicidae) (Muturi et al., 2016c). In *Cx. pipiens*, *Wolbachia* causes partial or complete cytoplasmic incompatibility (CI) between males and females infected by incompatible strains (Sinkins et al., 2005). It confers a relative fitness advantage to infected females allowing *Wolbachia* to rapidly invade host populations (Turelli and Hoffmann, 1991). *Wolbachia* (*Rickettsiaceae*) was detected in the mosquito samples in our study and was one of the dominant bacterial taxa identified in *Ar. Subalbatus* (93.81%) *and Ae. albopictus* (84.34%) from Sanya and *Ae. albopictus* (84.69%) from Dingan. It is unclear why *Wolbachia* was very low in *Cx. pallidothorax* (0.031% and 0.053%) from Dingan and Wenchang and *Cx. pipiens* (0.0057%) from Wuzhishan. At the genus level, especially in *Ae. albopictus* and *Cx. pipiens* mosquitoes from Wuzhishan, the unclassified taxa accounted for 60.30% and 99.14% (**Figure 2** and **S1**; **Table S3**). This might be due to variability in physiological conditions of individual mosquitoes or on habitat environmental conditions. Otherwise, most bacterial OTUs were sparsely distributed among individuals of the different mosquito species. This might be due to inter-individual variations in diet or genetic factors (Wang et al., 2011; Osei-Poku et al., 2012). These variations may indicate population and species level variation in vector competence. Certain bacterial species can increase (Apte-Deshpande et al., 2012) or reduce (Ramirez et al., 2012) vector susceptibility to pathogens.

There were differences in bacterial diversity and evenness between the mosquito species studied. The richness data (**Figure 5A**; **Table 2**) show that the *Ae. albopictus* from Ledong were significantly diverse and distributed compared to *Ae. albopictus* from Sanya. Significant differences in bacterial diversity and evenness between populations of the same mosquito species across different locations suggest that the sampling site environment is a key determinant of the bacterial profiles in mosquito guts. However, similar bacterial diversity and evenness between mosquito species across the four genera suggests that the mosquito midgut plays an important role in regulating the colonization and assembly of bacterial communities.

The β-diversity results showed that bacterial communities of mosquitoes vary geographically. The Bray-Curtis distances and the Weighted unifrac Beta diversity (**Figures 6A, B**) show that this variation in some mosquitoes resulted from the presence or absence of rare members, as well as some dominant genera. *Cx. pipiens* from Wuzhishan and *Ae. albopictus* from Tunchang were substantially different from other sample locations. In contrast, there was little separation between the microbiota of *Ae. albopictus* from Sanya and Dingan (**Figure 6A**; **Table S4**). Some members of the intraspecific core bacteriome of these mosquitoes were not the most abundant within the community, and their relative abundance varied among locations. A similar pattern of β-diversity variation, provided by the less-frequent members, was observed in another insect, include the *pine weevil*, *bark beetles*, *brown planthopper* and *Haemaphysalis longicornis* (Durand et al., 2015; Berasategui et al., 2016; Zhang et al., 2018; Zhang et al., 2019).

Midgut microbial composition and diversity are acquired through vertical inheritance and from the surrounding environment (Minard et al., 2013; Buck et al., 2016). These sources include food sources (e.g., nectar and blood meal), weather, and population growth. The environment of the sampling site is a key determinant of the bacteria that colonize the mosquitoes (Hansen et al., 2012). When the physiological conditions in the mosquito gut change, the pathogenic or commensal capacities of some bacteria may be influenced (Seaman et al., 2015; Short et al., 2017). Differences in food sources (Berasategui et al., 2016) may affect bacterial differences.

In summary, we characterized the midgut bacterial communities of six mosquito species in Hainan province, China. *Proteobacteria* and *Wolbachia* (*Rickettsiaceae*) were the major members of bacterial communities associated with most of the mosquito species. There were significant differences in the gut microbial composition between some species and substantial variation in the gut microbiota between individuals of the same mosquito species. There was a marked variation in different mosquito gut microbiota within the same location. These results might be useful in the identification of microbial communities that could be exploited for disease control.

## Data Availability Statement

The datasets presented in this study can be found in online repositories. The names of the repository/repositories and accession number(s) can be found below: https://datadryad.org/stash, https://datadryad.org/stash/share/VZY6nQMlADnSMgkzQ8iLuA2PtkPFooCFwzHBO8umhNA.

## Author Contributions

Conceptualization: LK. Data curation: YW, ZZ, QX. Formal analysis: ZZ, QX, AZ. Investigation: XK, YW, SL, YL, XS, XL. Methodology: XK, YW. Supervision: ZZ, QX. Writing original draft: XK, YW, MR, ZZ, QX. All authors contributed to the article and approved the submitted version.

## Funding

This work was supported by the National Key Plan for Scientific Research and Development of China (2019YFC1200504), the National Science and Technology Major Project (2018ZX10101004), and the National Natural Science Foundation of China (81560002, 81960002), Key Laboratory of Vector Biology and Pathogen Control of Zhejiang Province, Huzhou University (HUZUL201901).

## Conflict of Interest

The authors declare that the research was conducted in the absence of any commercial or financial relationships that could be construed as a potential conflict of interest.

## Supplementary Material

Supplementary Figure 1 Relative abundance of the top 43 bacterial genera in all samples from different study sites. DA = Dingan, HK = Haikou, LD = Ledong, LS = Ledong, SY = Sanya, TC = Tunchang, WC = Wenchang, WZS = Wuzhishan; AAL = *Aedes albopictus*, ASU = *Armigeres subalbatus*, CGE = *Culex gelidus*, AGA = *Aedes galloisi*, CPA = *Culex pallidothorax*, CPI = *Culex pipiens*.

Supplementary Figure 2 PCoA plot **(A–C)** based on an unweighted Unifrac distance matrix depicting differences in the composition of the gut microbiota from different locations. Colors represent community profiles of individual samples and location.
