# Supplementary figures and images for: Comparative Analysis of the Gut Microbiota of Adult Mosquitoes From Eight Locations in Hainan, China
Source: Front Cell Infect Microbiol. 2020 Dec 15;10:596750. doi: 10.3389/fcimb.2020.596750 (PMC7769952; doi:10.3389/fcimb.2020.596750)

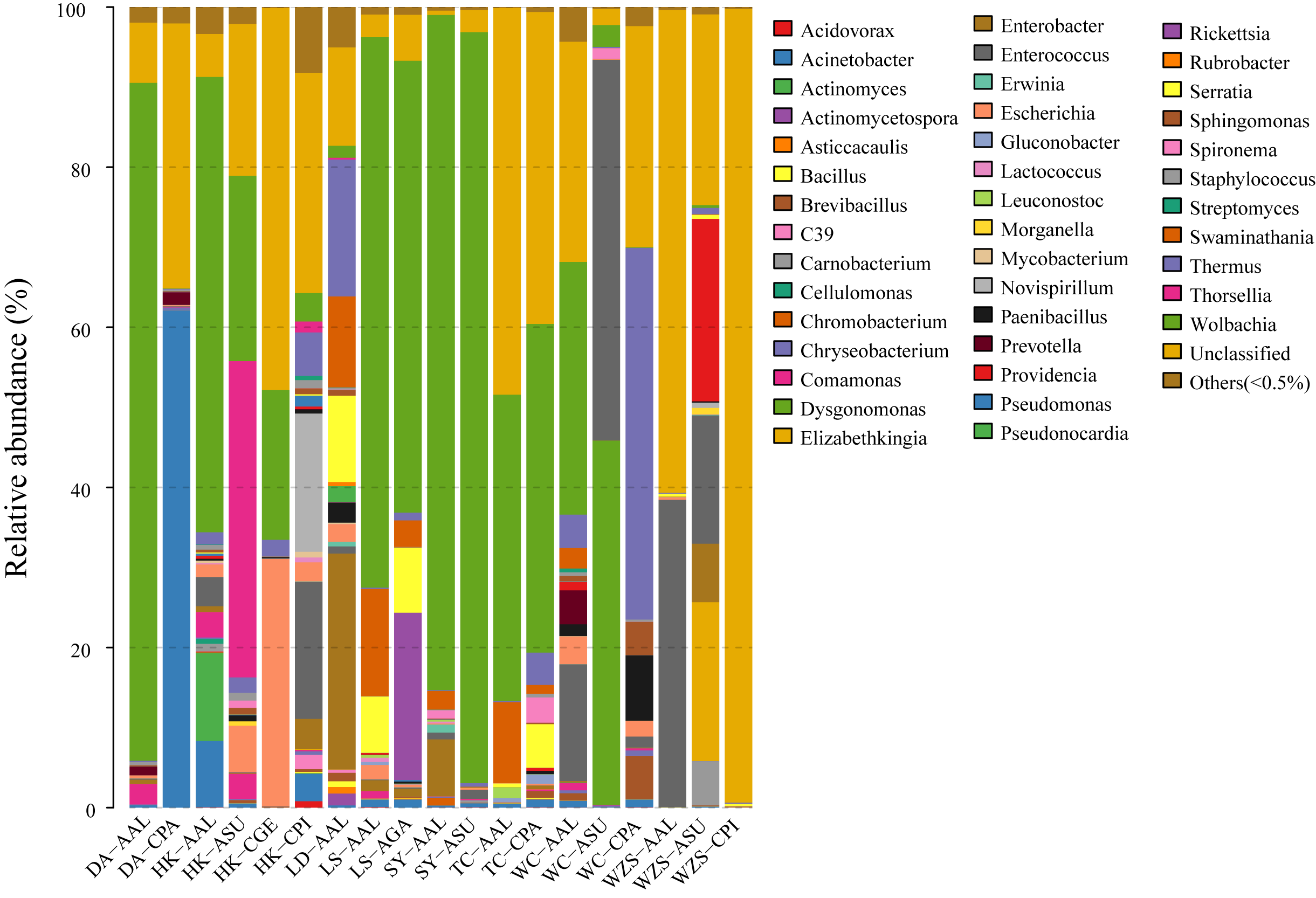

Supplement: Supplementary Figure 1 — Relative abundance of the top 43 bacterial genera in all samples from different study sites. DA = Dingan, HK = Haikou, LD = Ledong, LS = Ledong, SY = Sanya, TC = Tunchang, WC = Wenchang, WZS = Wuzhishan; AAL = Aedes albopictus, ASU = Armigeres subalbatus, CGE = Culex gelidus, AGA = Aedes galloisi, CPA = Culex pallidothorax, CPI = Culex pipiens. [file Image_1.tif]

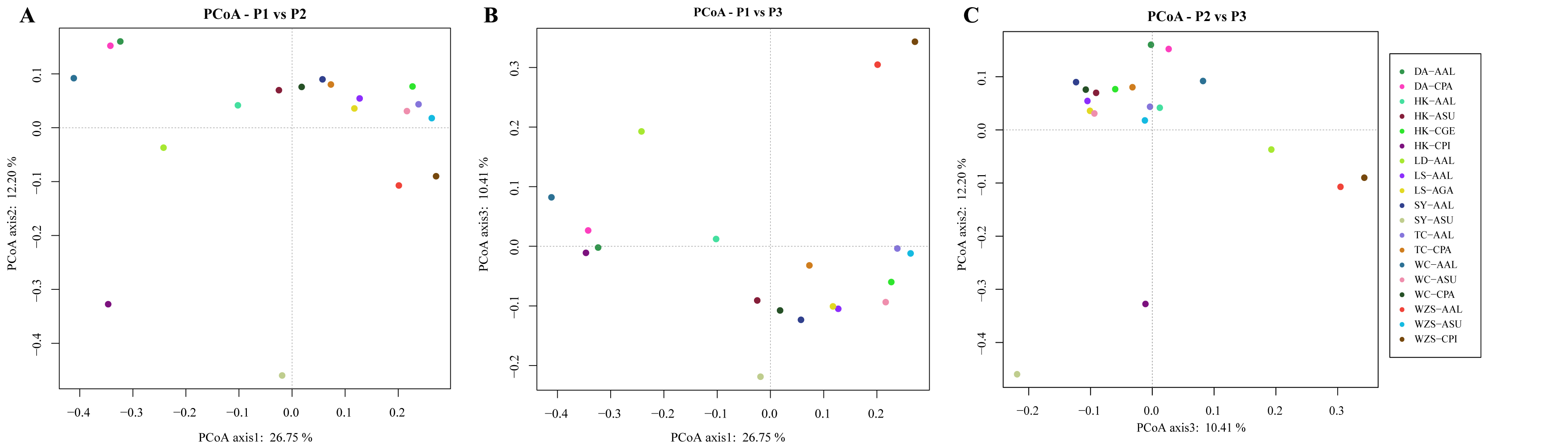

Supplement: Supplementary Figure 2 — PCoA plot (A–C) based on an unweighted Unifrac distance matrix depicting differences in the composition of the gut microbiota from different locations. Colors represent community profiles of individual samples and location. [file Image_2.tif]
